# Pulmonary rehabilitation for people with chronic obstructive pulmonary disease: A protocol for an overview of Cochrane reviews

**DOI:** 10.1097/MD.0000000000017129

**Published:** 2019-09-20

**Authors:** Zênia T.S. Araujo, Karla M.P.P. Mendonça, Bruma M.M. Souza, Tacito Z.M. Santos, Gabriela S.S. Chaves, Brenda N.G. Andriolo, Patricia A.M.S. Nogueira

**Affiliations:** a Laboratory of measures and evaluation in health; b Postgraduate Course in Physiotherapy of the Federal University of Rio Grande do Norte, Natal, Brazil; c University of New Hampshire, Durham, NH; d Laboratory of evaluation and respiratory intervention, Department of Physiotherapy; e School of Kinesiology and Health Science, York University, Toronto, Canada; f Cochrane Brazil, Center for Evidence-Based Health Studies and Technology Assessment in Health, São Paulo; g Laboratory of measures and evaluation in health, Postgraduate Course in Physiotherapy of the Federal University of Rio Grande do Norte, Natal, Brazil.

**Keywords:** chronic obstructive pulmonary disease, overview, pulmonary disease, pulmonary rehabilitation, therapeutic exercise

## Abstract

**Background:**

Pulmonary rehabilitation (PR) is an indispensable component in the nonpharmacological management of patients with chronic obstructive pulmonary disease (COPD) with significant improvements in quality of life and exercise capacity. It is strongly supported by systematic reviews (SR) as part of the treatment of these patients. However, it is not known which PR components are essential, such as duration, ideal locations, type and intensity of training, degree of supervision, adherence, cost-effectiveness challenge, and how long the program effects last. This overview aims to evaluate and describe different pulmonary rehabilitation interventions for individuals with COPD.

**Methods:**

Only systematic reviews of randomized controlled trials (RCTs) published in the Cochrane Database of Systematic Reviews will be included. The following results were analyzed: health-related quality of life, functional capacity, mortality, dyspnea, cost-effectiveness, and adverse events. The risk of bias will be assessed by the Risk of Bias in Systematic Reviews (ROBIS). The methodological quality will be analyzed through the Assessment of Multiple Systematic Reviews (AMSTAR-2). We will use the evaluations of the Classification of Recommendations, Evaluation, Development and Evaluation (GRADE) of the authors of the included systematic reviews. The screening of systematic reviews, eligibility evaluation, data extraction, methodological quality, and quality of evidence will be performed in pairs by independent reviewers. The results that have been reported in the included reviews will be summarized in an “Overview of Reviews” table. The main conclusions about the effects of the interventions studied in the included reviews will be summarized and organized in clinically meaningful categories.

**Results:**

The article in this overview will be submitted for publication in a peer-reviewed journal. The results will also be included in a doctoral thesis and disclosed in medical conferences.

**Conclusions:**

We expect to compile evidence from multiple systematic reviews of pulmonary rehabilitation in people with COPD in an accessible and useful document.

**Registration number PROSPERO:**

CRD42019111564.

## Abstract 2

Supplemental Digital Content is available in the text

## 1 Introduction

Chronic obstructive pulmonary disease (COPD) is a frequent disease, determined by constant respiratory symptoms and chronic airflow limitation. It is clinically determined due to exacerbations, comorbidities, and symptoms, such as: dyspnoea, cough, and/or expectoration. Chronic airflow limitation is a characteristic of COPD and is caused by airway and/or alveolar abnormalities.^[1–4]^ The diagnosis requires confirmation by spirometry (FEV_1_/FVC ≤ 70% post-bronchodilator or FEV_1_/FVC ≤ 70% and FEV_1_ < 80% pre-bronchodilator—where post-bronchodilator testing is not possible) and history of exposure to particulate matter or harmful gases.^[5–10]^

The data indicate worldwide a high prevalence of COPD with projections of increase over the next 30 years, with estimated annual mortality of >45 million people.^[1,11]^ As a prevalent disease, COPD is also associated with comorbidities, with a high degree of disability and with a consequent financial burden, implying in significant consequences for health and the economy.^[12–15]^ These factors have a major impact on health and economy and make it a major challenge for managers.^[16,17]^

Thus, considering the current scenario of pathophysiological and functional changes in COPD, we have been looking for more effective pharmacological and non-pharmacological strategies in the management of these patients. Within these approaches, pulmonary rehabilitation programs,^[1,18,19]^ according to the American Thoracic Society (ATS) and the European Respiratory Society (ERS), stand out as “a comprehensive intervention based on a comprehensive patient assessment followed by patient-tailored therapies, which include, but are not limited to, physical training, education, and behavior change, aimed at improving the physical and psychological condition of people with chronic respiratory disease and promoting adherence long-term health-enhancing behaviors.”^[19]^

Emphasis is given to the physical training that can be performed in groups, but with individualized sessions that involve aerobic, resistance, interval or continuous exercises, resistance/strength, flexibility, neuromuscular electrical stimulation, exercises that involve the upper and lower limbs, in addition to inspiratory muscle training.^[1,9,19]^ Pulmonary rehabilitation can be performed in different settings, such as: hospital, outpatient clinic, or home.^[18–21]^ The evidence^[9,22,23]^ points to the inclusion in the rehabilitation of these patients with COPD at all levels of severity of impairment of pulmonary function, especially in moderate to severe pulmonary function.

The inclusion of people with COPD in these programs should be based on symptoms and functional limitations, rather than just on the severity of lung impairment,^[1,19,24]^ such as: exertional dyspnea secondary to ventilatory impairment,^[25]^ low levels of physical activity and depression,^[26–28]^ comorbid conditions such as cardiovascular and cerebrovascular diseases, endocrine and metabolic disorders, psychiatric and neurological disorders, gastrointestinal disorders, musculoskeletal disorders,^[29]^ exacerbations of the disease, and impairment of quality of life.^[1,6,9]^

Thus, the evidence indicates the following physiological benefits of the physical training component in pulmonary rehabilitation in patients with COPD: decrease in circulating inflammatory markers,^[30–32]^ better supply of oxygen to respiratory and peripheral muscles,^[33]^ increased carbon monoxide diffusion capacity, and effort tolerance.^[31]^

Therefore, the benefits of pulmonary rehabilitation in patients with COPD are related to clinical improvement directly reflected in health-related quality of life, dyspnea, fatigue, emotional function, and exercise capacity according to Cochrane systematic review and meta-analysis,^[18]^ as well as the current clinical guidelines.^[1,6,19]^

In recent years the number of Cochrane systematic reviews has been increasing, which addresses pulmonary rehabilitation in patients with COPD, and directly reflects the assistance model focused on approaches that aim to modify the behavior of this population. These Cochrane reviews point to different models in providing care that involves the traditional inpatient or outpatient model as alternative models in the community or at home.^[1,6,18,23]^

Despite all the advances in pulmonary rehabilitation, there are still issues to be improved, as: to increase patient access to rehabilitation programs around the world; to understand effects during hospitalization due to exacerbation and/or after early exacerbation (within 1 month of exacerbation); benefits in the early stage of COPD (mild disease); alternative models of pulmonary rehabilitation (use of new technologies, telerreabilitation, home rehabilitation, use of minimal equipment or without equipment, self-management); degree of supervision; intensity of exercises; ideal time, and duration of the effects of rehabilitation.^[18,34]^

Understanding these issues can be useful in guiding therapeutic and policy decisions (e.g., health-related quality of life impacts, functional capacity, cost-effectiveness, adverse events) in a single, scientifically accessible document to provide a “friendly front end,” so that the reader does not have to assimilate the data from separate systematic reviews.^[35]^ Thus, this overview aims to summarize the evidence from the different available models of pulmonary rehabilitation interventions for COPD patients, to identify evidence gaps in the current literature to inform about new titles for systematic review of pulmonary rehabilitation, and to describe pulmonary rehabilitation interventions that patients with COPD.

## 2 Methods

It is an overview protocol that follows the recommendations of the Cochrane Handbook for Systematic Reviews of Interventions.^[36]^ This protocol was recorded in the Prospective International Registry of Systematic Review (PROSPERO), registration number CRD42019111564. (https://www.crd.york.ac.uk/prospero/display_record.php?RecordID=111564).

### 2.1 Criteria for inclusion of revisions

#### 2.1.1 Types of study

For this overview, only systematic reviews of randomized controlled trials (RCTs) for pulmonary rehabilitation in people with COPD, published in the Cochrane Database of Systematic Reviews, will be included. This overview seeks to assess the evidence published in Cochrane original systematic reviews and will not attempt to update these reviews. However, specific information on intervention components can be requested from test reports and individual researchers.

##### 2.1.1.1 Inclusion criteria

For the purposes of this overview, systematic reviews evaluating pulmonary rehabilitation including physical training (e.g., aerobic exercise, resisted exercise or aerobic, and resisted exercise) will be included; educational component and/or psychological support such as intervention. Supervised or unsupervised interventions will be included in a rehabilitation center, hospital, or home.^[1,3,9]^

##### 2.1.1.2 Exclusion criteria

We will exclude reviews of non-pharmacological treatments and treatment devices that are beyond the scope of this overview.

#### 2.1.2 Participants/population

Cochrane reviews of people with COPD diagnosed based on clinical and/or spirometric criteria^[1,2,9]^ will be included. Adults (18 years of age or older) without any restrictions based on the severity of the disease or in the exacerbated state. In this overview we will consider the standardized by review authors from “valid” concepts.

#### 2.1.3 Intervention

Systematic reviews that evaluated pulmonary rehabilitation including physical training (e.g., aerobic exercise, resisted exercise or aerobic, and resisted exercise); educational component and/or psychological support such as intervention. Supervised or unsupervised interventions will be included in a rehabilitation center, hospital, or home.^[1,3,9]^

#### 2.1.4 Comparator (s)/control

Any control considered for comparison in individual systematic reviews. This includes other treatments, no treatment, or placebo.

#### 2.1.5 Outcomes

##### 2.1.5.1 Primary outcomes

- Health-related quality of life (HRQoL) (measured by Saint George's Respiratory Questionnaire, Clinical COPD Questionnaire, 36-Item Short Form Health Survey questionnaire, COPD Assessment Test, or any validated instrument);
- Functional capacity (measured by cardiopulmonary exercise test—CPET; shuttle walk tests—SWTS; 6 minute walk test—6MWT, or any other validated instrument);
- Mortality.

##### 2.1.5.2 Secondary outcomes

- Dyspnea (as measured by MRC, Borg, or any other validated instrument);
- Cost-effectiveness;
- Adverse events (hospitalizations, absenteeism, at work, exacerbations).

### 2.2 Research methods to identify revisions

The searches will be conducted in the Cochrane Systematic Reviews Database (CDSR), in the Cochrane Library. The search strategy is presented in Supplementary Digital Content (Appendix 1). Non-cochrane reviews will not be considered or redeemed for this overview. We will note when the included reviews are outdated, whether new relevant studies have been published, and whether there is any relevant intervention for which a systematic review has not yet been published. However, updates to systematic reviews or new systematic reviews should not be performed within the overview. We will not apply date or language restrictions. All protocols for revisions will be noted in the “Studies awaiting evaluation” section for possible inclusion in future updates of this overview.

### 2.3 Collection and analysis of data

#### 2.3.1 Selection of revisions

Two authors of this overview (ZTSA and TZMS) will independently evaluate all revisions retrieved through the eligibility survey using the criteria listed above under criteria for considering revisions for inclusion. We will resolve all conflicts through discussions to reach a by a third party.

#### 2.3.2 Extraction and management of data

Data extraction from each included revision will be performed independently by 2 authors (ZTSA and TZMS) using Review Manager 5.3.5 (the Cochrane Collaboration, London, United Kingdom).^[37]^ Possible disagreements will be resolved by a third author (PAMSN) of the overview. From each of the included reviews, relevant data such as the number of trials included, the number of participants included, the date of the last survey, and the inclusion and exclusion criteria will be extracted. The characteristics such as population, intervention (pulmonary rehabilitation) and dose (frequency/intensity), adherence, update check, comparison, control description, outcomes, and limitations of the review will also be presented in a Table 1 containing “Characteristics of included reviews.”

**Table 1:**
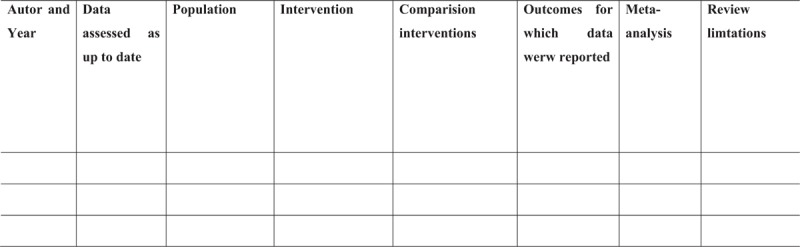
Characteristics of included reviews.

### 2.4 Evaluation of the methodological quality of the included revisions

#### 2.4.1 Quality of included revisions

Two authors of the overview (ZTSA and GSSC) will independently evaluate the methodological quality in each review included to assess whether they met the criteria specified in the “Assessment of Various Systematic Reviews” (AMSTAR-2).^[38]^ Disagreements will be resolved through discussion between them and with the arbitration of a third general author (PAMSN) if necessary. The results of the methodological quality assessment of the included reviews will be included in an additional Table 2.

**Table 2:**
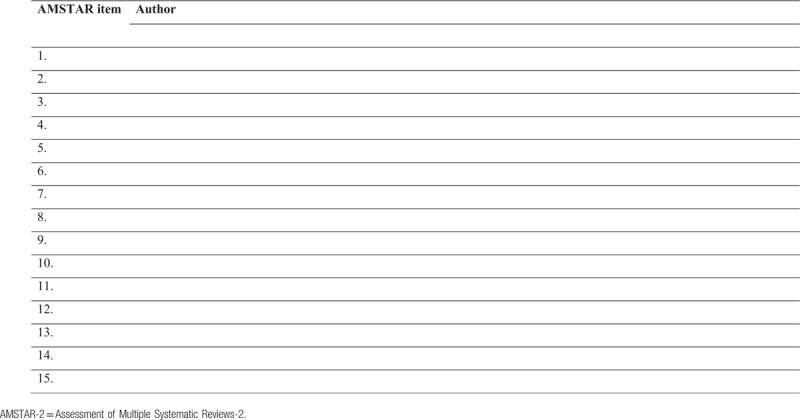
Methodological quality assessment of included reviews using AMSTAR-2.

#### 2.4.2 Risk assessment of bias

Two review authors (ZTSA and GSSC) will independently assess the risk of bias of the included revisions using the bias risk tool in systematic reviews (ROBIS).^[39]^ We will present in a Table 3 the assessment of individual ROBIS items or domains (along with justification for judgments for each evaluation—relevance, identification of potential bias risks during the review process, and general bias risk).

**Table 3:**
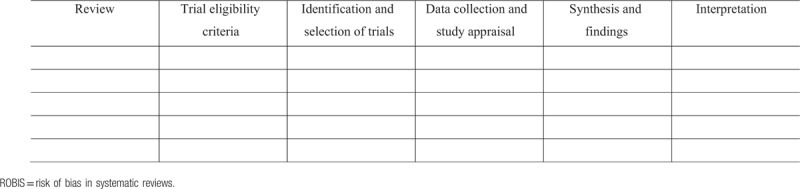
ROBIS assessment.

Risk of bias evaluation will be used to conduct sensitivity analyzes, but we will not rule out revisions based on bias assessment risk. We will summarize this information in accordance with the guidelines provided in the Cochrane Handbook for Systematic Reviews of Interventions.^[40]^

#### 2.4.3 Quality of evidence in included reviews

The strength of the evidence or the overall quality of the evidence provided in the included reviews will be evaluated using the GRADE approach as well as the GRADEpro Guideline Development Tool [Software]. McMaster University, (developed by Evidence Prime, Inc.), Ontario, Canada.^[41,42]^

This evaluation will be performed independently by 2 overview authors (ZTSA and GSSC) to assess the quality of evidence throughout the studies for each important outcome. Any disagreements will be resolved through discussion in the overview authors team. The results will be represented in the “Summary of findings” Table 4.

**Table 4:**
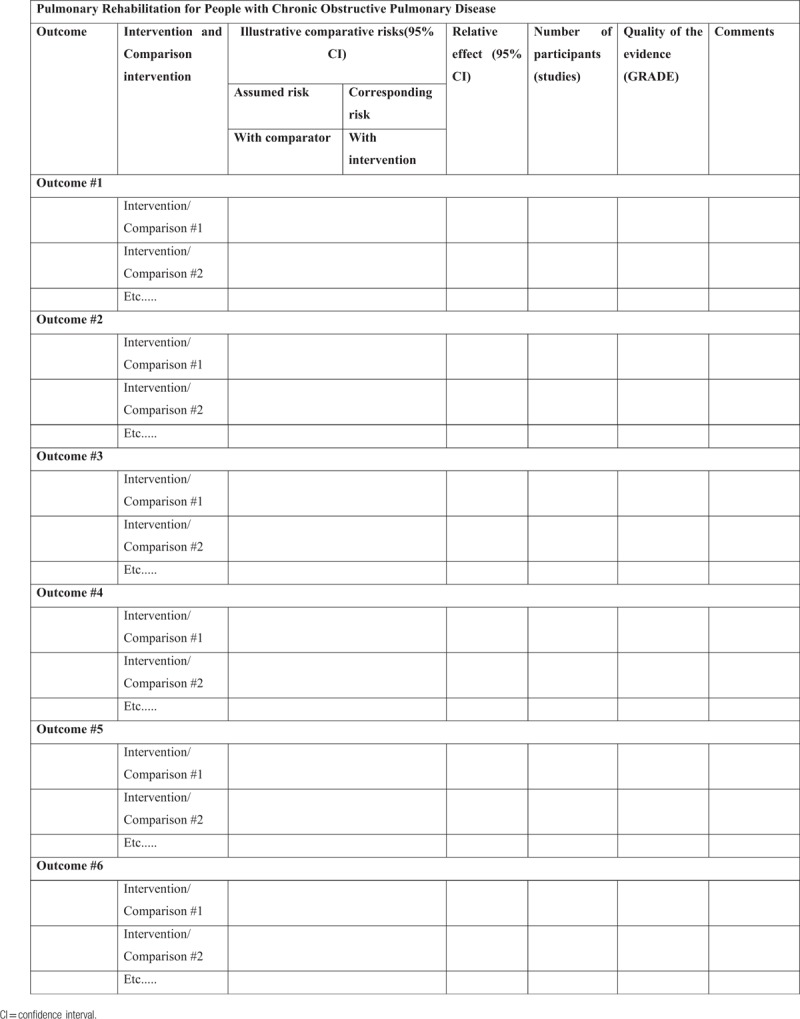
Summary of findings from included reviews.

#### 2.4.4 Overview review table

The results reported in the included reviews will be summarized in an “Overview reviews” table by result and then by comparison. The table should include beneficial and detrimental results, frequency or severity of these outcomes in the control groups, estimates of relative and absolute effects of interventions, bias risk indications (which may vary by outcome and comparison), and comments if necessary.

#### 2.4.5 Measures of the treatment effect

The main conclusions about the effects of the interventions studied in the included reviews will be summarized and organized around clinically significant categories (e.g., types of interventions or types of outcomes). For this, the results of included studies will be interpreted by the reports made in the reviews, without having to resort to the original data of the study. If the data are reported as a mean difference (MDs) or as an absolute or relative change score, appropriate scales (when possible) will be considered to determine if this was clinically significant. For data presented as standardized mean difference (SMD), with or without 95% confidence intervals (CI) or level of significance (*P* value), Cohen interpretation^[43]^ will be useful to define the effect size. For example, using SMD, the effect size will be classified as small (SMD 0.2–0.5), moderate (SMD 0.5–0.8), or large (SMD >0.8).

#### 2.4.6 Problems in the analysis unit

It is hoped that Cochrane reviews have already addressed these issues. However, if not, it will be considered to contact the original authors for clarification on unit analysis issues that were not reported in the intervention review.

#### 2.4.7 Dealing with lost data

Data from missed outcomes of intervention reviews are either because the review authors did not report on these or found no evidence will be considered as “no evidence.” The data from the original test reports will not be extracted if this data has not been collected in the intervention reviews.

### 2.5 Synthesis of data

The unit of analysis for this overview are systematic reviews (not individual trials). Thus, the PICO elements will be tabulated at the revision level. Results tables will include effect estimates, with 95% confidence intervals (CIs), and measures of heterogeneity/risk of bias, as appropriate. Estimates of effect of included systematic reviews, categorized by intervention and primary and secondary outcomes, will be extracted and presented in tables and figures. The narrative descriptions of the estimates of the effects of the included revisions will be structured according to the risk of systematic review bias and GRADE evaluation.

Choosing the effect estimate for summary and tabulation will depend on the results reported in several revisions. We intend to standardize the reported results if a result is expressed differently between reviews. We will standardize risk indices (RRs) or odds ratios (ORs) for dichotomous outcomes. We will standardize as differences (MDs) or differences of standardized means (SMDs) using equations published in the Cochrane Handbook for Systematic Reviews of Interventions for continuous results.^[40]^

The exact method chosen for graphical display will depend on the number of studies available for each specific result. Review Manager 5^[37]^ will be used to generate standardized effect charts and use them to graphically present the results, with each revision representing a line in the forest plot.

We will discuss the limitations of currently available evidence regarding heterogeneity of inclusion criteria for each review, consistency of effect size for each intervention, and consistent use of outcome measures. We will identify gaps in the current evidence base and make recommendations for future research.

Although the sequence of tables has been planned we know that it depends on the availability and how the effect estimates will be presented by the included reviews.

#### 2.5.1 Subgroup analysis and investigation of heterogeneity

If possible, a subgroup analysis of separate review data will be performed, grouped by differences in the scope of the review. Such as disease severity (stable vs exacerbation); age, and location where pulmonary rehabilitation was offered (hospital, rehabilitation center, home).

## 3 Ethics and dissemination

Ethical approvals and patient consent are not required, as this overview will be based on a published systematic review. No primary data will be collected. The article in this overview will be submitted for publication in a peer-reviewed journal. The results will also be included in a doctoral thesis and published in scientific conferences.

## Author contributions

**Conceptualization:** Zenia Trindade de Souto Araujo, Brenda Nazare Gomes Andriolo, Patricia Angelica Miranda Silva Nogueira.

**Data curation:** Zenia Trindade de Souto Araujo.

**Formal analysis:** Zenia Trindade de Souto Araujo, Gabriela Suellen da Silva Chaves.

**Investigation:** Zenia Trindade de Souto Araujo, Patricia Angelica Miranda Silva Nogueira.

**Methodology:** Zenia Trindade de Souto Araujo, Karla Morganna Pereira Pinto Mendonça, Tacito Zaildo Morais Santos, Gabriela Suellen da Silva Chaves.

**Project administration:** Zenia Trindade de Souto Araujo, Patricia Angelica Miranda Silva Nogueira.

**Resources:** Zenia Trindade de Souto Araujo, Tacito Zaildo Morais Santos, Patricia Angelica Miranda Silva Nogueira.

**Software:** Zenia Trindade de Souto Araujo, Gabriela Suellen da Silva Chaves.

**Supervision:** Karla Morganna Pereira Pinto Mendonça, Patricia Angelica Miranda Silva Nogueira.

**Validation:** Karla Morganna Pereira Pinto Mendonça, Bruma Morganna Mendonça Souza, Brenda Nazare Gomes Andriolo, Patricia Angelica Miranda Silva Nogueira.

**Visualization:** Karla Morganna Pereira Pinto Mendonça, Brenda Nazare Gomes Andriolo, Patricia Angelica Miranda Silva Nogueira.

**Writing – original draft:** Zenia Trindade de Souto Araujo, Karla Morganna Pereira Pinto Mendonça, Bruma Morganna Mendonça Souza, Tacito Zaildo Morais Santos, Gabriela Suellen da Silva Chaves, Brenda Nazare Gomes Andriolo, Patricia Angelica Miranda Silva Nogueira.

**Writing – review & editing:** Zenia Trindade de Souto Araujo, Karla Morganna Pereira Pinto Mendonça, Bruma Morganna Mendonça Souza, Tacito Zaildo Morais Santos, Gabriela Suellen da Silva Chaves, Brenda Nazare Gomes Andriolo, Patricia Angelica Miranda Silva Nogueira.

Karla Morganna Pereira Pinto de Mendonça orcid: 0000-0001-5734-3707.

Bruma Morganna Mendonça de Souza orcid: 0000-0002-9766-8255.

Tácito Zaildo de Morais orcid: 0000-0002-9495-7078.

Gabriela Suellen da Silva Chaves orcid: 0000-0002-7737-8015.

Brenda Nazare Gomes Andriolo orcid: 0000-0001-6343-666X.

Patrícia Angélica de Miranda Silva Nogueira orcid: 0000-0002-3763-2410.

Zenia Trindade de Souto Araujo orcid: 0000-0003-3447-6990.

## Supplementary Material

Supplemental Digital Content

## References

[1] Global Initiative for Chronic Obstructive Lung Disease (GOLD). The global strategy for diagnosis, management and prevention of COPD 2019. Available at: https://goldcopd.org/wp-content/uploads/2018/11/GOLD-2019-v1.7-FINAL-14Nov2018-WMS.pdf (accessed prior to February 22, 2019).

[2] Papi A Rabe KF Rigau D Management of COPD exacerbations: a European Respiratory Society/American Thoracic Society guideline. Eur Respir J 2017;49:1–6. 10.1183/13993003.00791-2016 28298398

[3] Alison JÁ McKeough ZJ Johnston K Australian and New Zealand pulmonary rehabilitation guidelines. Respirology 2017;22:800–19. 28339144 10.1111/resp.13025

[4] Wedzicha JA Miravitlles M Hurst JR Management of COPD exacerbations: a European Respiratory Society/American Thoracic Society guideline. Eur Respir J 2017;49:pii: 1600791. 10.1183/13993003.00791-2016 28298398

[5] Yang IA Brown JL George J The COPD-X Plan: Australian and New Zealand Guidelines for the management of Chronic Obstructive Pulmonary Disease 2018. Version 2.56, December 2018. Available at: https://copdx.org.au/wp-content/uploads/2019/02/COPDX-V2-56-Dec-2018-Web.pdf Accessed prior to May 31, 2019

[6] National Institute for Health and Care Excellence (NICE). Chronic obstructive pulmonary disease in over 16s: diagnosis and management. NICE guideline; 2019. Available at: https://www.nice.org.uk/guidance/ng115 (Accessed prior to July 26, 2019). 31211541

[7] Calle Rubio M Casamor R Miravitlles M Identification and distribution of COPD phenotypes in clinical practice according to Spanish COPD Guidelines: the FENEPOC study. Int J Chron Obstruct Pulmon Dis 2017;12:2373–83. 28848338 10.2147/COPD.S137872 PMC5557116

[8] Forum of International Respiratory Societies (FIRS). The Global Impact of Respiratory Disease – Second Edition. Sheffield, European Respiratory Society; 2017.

[9] Celli BR Decramer M Wedzicha JA An Official American Thoracic Society/European Respiratory Society Statement: research questions in chronic obstructive pulmonary disease. Am J Respir Crit Care Med 2015;191:e4–27. 25830527 10.1164/rccm.201501-0044ST

[10] Miravitlles M Calle M Soler-Cataluña JJ Clinical phenotypes of COPD: identification, definition and implications for guidelines. Arch Bronconeumol 2012;48:86–98. 22196477 10.1016/j.arbres.2011.10.007

[11] Rossi A Butorac-Petanjek B Chilosi M Chronic obstructive pulmonary disease with mild airflow limitation: current knowledge and proposal for future research – a consensus document from six scientific societies. Int J Chron Obstruct Pulmon Dis 2017;12:2593–610. 28919728 10.2147/COPD.S132236 PMC5587130

[12] Soriano JB Abajobir AA Abate KH Global, regional, and national deaths, prevalence, disability-adjusted life years, and years lived with disability for chronic obstructive pulmonary disease and asthma, 1990-2015: a systematic analysis for the Global Burden of Disease Study 2015. Lancet Respir Med 2017;5:691–706. 28822787 10.1016/S2213-2600(17)30293-X PMC5573769

[13] Rosenberg SR Kalhan R Mannino DM Epidemiology of chronic obstructive pulmonary disease: prevalence, morbidity, mortality, and risk factors. Semin Respir Crit Care Med 2015;36:457–69. 26238634 10.1055/s-0035-1555607

[14] Diaz-Guzman E Mannino DM Epidemiology and prevalence of chronic obstructive pulmonary disease. Clin Chest Med 2014;35:7–16. 24507833 10.1016/j.ccm.2013.10.002

[15] Smith MC Wrobel JP Epidemiology and clinical impact of major comorbidities in patients with COPD. Int J Chron Obstruct Pulmon Dis 2014;27:871–88. 10.2147/COPD.S49621 PMC4154888 25210449

[16] Negewo NA Gibson PG McDonald VM COPD and its comorbidities: impact, measurement and mechanisms. Respirology 2015;20:1160–71. 26374280 10.1111/resp.12642

[17] Blasi F Cesana G Conti S The clinical and economic impact of exacerbations of chronic obstructive pulmonary disease: a cohort of hospitalized patients. PLoS One 2014;27:e101228. 10.1371/journal.pone.0101228 PMC4074190 24971791

[18] McCarthy B Casey D Devane D Pulmonary rehabilitation for chronic obstructive pulmonary disease. Cochrane Database Syst Rev 2015 CD003793. 25705944 10.1002/14651858.CD003793.pub3 PMC10008021

[19] Spruit MA Singh SJ Garvey C An Official American Thoracic Society/European Respiratory Society statement: key concepts and advances in pulmonary rehabilitation e an executive summary. Am J Respir Crit Care Med 2013;188:13–64. 10.1164/rccm.201309-1634ST 24127811

[20] Nolan CM Kaliaraju D Jones SE Home versus outpatient pulmonar rehabilitation in COPD: a propensity-matched cohort study. Thorax 2019;0:pii: thoraxjnl-2018-212765. 10.1136/thoraxjnl-2018-212765 31278173

[21] Holland AE Mahal A Hill CJ Home-based rehabilitation for COPD using minimal resources: a randomised, controlled equivalence trial. Thorax 2017;72:57–65. 27672116 10.1136/thoraxjnl-2016-208514 PMC5329049

[22] Sahin H Naz I Varol Y Is a pulmonar rehabilitation program effective in COPD patients with chronic hypercapnic failure? Expert Ver Respir Med 2016;10:593–8. 10.1586/17476348.2016.1164041 26954769

[23] Puhan MA Gimeno-Santos E Cates CJ Pulmonary rehabilitation following exacerbations of chronic obstructive pulmonary disease. Cochrane Database Syst Rev 2016;12:CD005305. 27930803 10.1002/14651858.CD005305.pub4 PMC6463852

[24] Bolton CE Bevan-Smith EF Blakey JD British Thoracic Society Pulmonary Rehabilitation Guideline Development Group; British Thoracic Society Standards of acre Committee. British Thoracic Society guideline on pulmonary rehabilitation in adults. Thorax 2013;68:ii1–30. 23880483 10.1136/thoraxjnl-2013-203808

[25] Kagawa H Miki K Kitada S Dyspnea and the varying pathophysiologic manifestations of chronic obstructive pulmonary disease evaluated by cardiopulmonary exercise testing with arterial blood analysis. Front Physiol 2018;9:1293. 30333757 10.3389/fphys.2018.01293 PMC6176099

[26] Mihaltan F Adir Y Antczak A Importance of the relationship between symptoms and self-reported physical activity level in stable COPD based on the results from the SPACE study. Respir Res 2019;20:89. 31088560 10.1186/s12931-019-1053-7 PMC6518503

[27] Odackal J Lyons G Harris D Depressive symptoms are associated with self-reported physical limitations that are activity dependent in a cross-sectional analysis of subjects with chronic obstructive pulmonary disease. COPD 2019 1–7. 10.1080/15412555.2019.1634684 31298042

[28] Watz H Pitta F Rochester CL An official European Respiratory Society statement on physical activity in COPD. Eur Respir J 2014;44:1521–37. 25359358 10.1183/09031936.00046814

[29] Yin H Yin S Lin Q Prevalence of comorbidities in chronic obstructive pulmonary diseasepatients: a meta-analysis. Medicine (Baltimore) 2017;96:e6836. 28489768 10.1097/MD.0000000000006836 PMC5428602

[30] Di Raimondo D Tuttolomondo A Buttà C Metabolic and anti-inflammatory effects of a home-based programme of aerobic physical exercise. Int J Clin Pract 2013;67:1247–53. 24246205 10.1111/ijcp.12269

[31] Garcia-Aymerich J Serra I Gómez FP Physical activity and clinical and functional status in COPD. Chest 2009;136:62–70. 19255291 10.1378/chest.08-2532

[32] Handschin C Spiegelman BM The role of exercise and PGC1α in inflammation and chronic disease. Nature 2008;454:463–9. 18650917 10.1038/nature07206 PMC2587487

[33] Rabinovich RA Ardite E Troosters T Reduced muscle redox capacity after endurance training in patients with chronic obstructive pulmonary disease. Am J Respir Crit Care Med 2001;164:1114–8. 11673195 10.1164/ajrccm.164.7.2103065

[34] Vercammen-Grandjean C Schopfer DW Zhang N Participation in pulmonary rehabilitation by veterans health administration and medicare beneficiaries after hospitalization for chronic obstructive pulmonary disease. J Cardiopulm Rehabil Prev 2018;38:406–10. 30252780 10.1097/HCR.0000000000000357

[35] Becker LA Oxman AD Chapter 22: Overviews of reviews. In: Higgins JPT Green S (editors), Cochrane Handbook for Systematic Reviews of Interventions Version 5.1.0 (updated March 2011). The Cochrane Collaboration, 2011. Available at: www.handbook.cochrane.org.

[36] Pollock M Fernandes RM Becker LA Higgins JPT Thomas J Chandler J Chapter V: overviews of reviews. Draft version (8 October 2018) for inclusion. Cochrane Handbook for Systematic Reviews of Interventions. London: Cochrane; 2018;Accessed August 2, 2019.

[37] Review Manager (RevMan) [Computer program]. The Cochrane Collaboration. Version 5.3. Copenhagen: The Nordic Cochrane Centre; 2014.

[38] Shea BJ Reeves BC Wells G AMSTAR 2: a critical appraisal tool for systematic reviews that include randomised or non-randomised studies of healthcare interventions, or both. BMJ 2017;358:j4008. 28935701 10.1136/bmj.j4008 PMC5833365

[39] Whiting P Savović J Higgins JP ROBIS: a new tool to assess risk of bias in systematic reviews was developed. J Clin Epidemiol 2016;69:225–34. 26092286 10.1016/j.jclinepi.2015.06.005 PMC4687950

[40] Chandler J Higgins JPT Deeks JJ Chapter 1: Introduction. In: Higgins JPT, Churchill R, Chandler J, Cumpston MS (editors), Cochrane Handbook for Systematic Reviews of Interventions Version 5.2.0 (updated February 2017), Cochrane; 2017. Available from Cochrane Community.

[41] Atkins D Eccles M Flottorp S Systems for grading the quality of evidence and the strength of recommendations I: Critical appraisal of existing approaches The GRADE Working Group. BMC Health Serv Res 2004;4:1–7. 15615589 10.1186/1472-6963-4-38 PMC545647

[42] GRADEpro GDT: GRADEpro Guideline Development Tool [Software]. McMaster University; 2015 (developed by Evidence Prime, Inc.). Available from gradepro.org.

[43] Cohen J Statistical Power Analysis for the Behavioral Sciences. New York, NY: Routledge Academic; 1988.

