# Supplementary material for: Pulmonary rehabilitation for people with chronic obstructive pulmonary disease: A protocol for an overview of Cochrane reviews
Source: Medicine (Baltimore). 2019 Sep 20;98(38):e17129. doi: 10.1097/MD.0000000000017129 (PMC6756730; doi:10.1097/MD.0000000000017129)
Supplement: Supplemental Digital Content [file medi-98-e17129-s001.docx]

**APPENDICES**

**Appendix 1. Search strategy**

1. MeSH descriptor Pulmonary Disease, Chronic Obstructive explode all trees
2. MeSH descriptor Bronchitis, Chronic
3. COPD: MISC1
4. (COPD OR COAD OR COBD OR AECOPD): TI,AB,KW
5. #1 OR #2 OR #3 OR #4
6. MeSH descriptor Rehabilitation explode all trees
7. MeSH descriptor Rehabilitation Centers explode all trees
8. MeSH descriptor Hospitals, Rehabilitation explode all trees
9. MeSH descriptor Exercise Therapy explode all trees
10. MeSH descriptor Exercise explode all trees
11. MeSH descriptor Physical Therapy Modalities explode all trees
12. # 6 OR #7 OR #8 OR #9 OR #10 OR #11
13. #12 AND #5

[Limited to Cochrane Database of Systematic Reviews]
